# Surveillance Data Use Through the Fleming Fund: Informing Action on Antimicrobial Resistance in Low- and Middle-Income Countries

**DOI:** 10.1093/cid/ciag520

**Published:** 2026-10-19

**Authors:** Archie Drake, Alesia Ofori, Amy Rahmadanti, Ankur Gupta-Wright, Areeya Disratthakit, Ayodele Majekodunmi, Benjamin Kissi Sasu, N Claire Gordon, Claudia Parry, Collins Mitambo, Deborah Nanjebe, Desrina Sitompul, Didem Torumkuney, Evelyn Wesangula, Freddy Eric Kitutu, Heidi Hopkins, Jacob Wildfire, Japheth A Opintan, Jennifer Armitage, Joseph Sam Kanu, Kate Macintyre, Kriti Khurana, Lamiaa Shehata, Manish Pathak, Megha Raj Banjara, Milena von und zur Mühlen, Muhammad Taimoor Kayani, Muhammad Usman Zaheer, Mwapu Ndahi, Nandini Shetty, Nicholas Feasey, Nigel French, Nimesh Poudyal, Nure Alam Siddiky, Paul Turner, Pem Chuki, Qadeer Ahsan, Rebecca Maudling, Ritu Amatya, Satyajit Sarkar, Syed Abbas, Tamalee Roberts, Tauqeer Mustafa, Tim Shorten, Titha Dzowela, Toby Leslie, Tochi Okwor, Yu Nandar Aung

**Affiliations:** Itad Ltd, Brighton, United Kingdom; Independent Consultant, Dar es Salaam, Tanzania; Itad Ltd, Brighton, United Kingdom; Independent Researcher, United Kingdom; Directorate of Quality of Referral Health Service, Ministry of Health, South Jakarta, Indonesia; Itad Ltd, Brighton, United Kingdom; Department of Infectious Disease, Imperial College London, London, United Kingdom; Department of Infectious Diseases and Tropical Medicine, University of Heidelberg, Heidelberg, Germany; Department of Infectious Diseases, Bristol NHS Trust, Bristol, United Kingdom; MODRA, Mahidol Oxford Tropical Medicine Research Unit, Bangkok, Thailand; Ajisefini Consulting, Abuja, Nigeria; National Food Safety Laboratory, Veterinary Services Department, Ministry of Food and Agriculture, Accra, Ghana; Rare and Imported Pathogens Laboratory, UK Health Security Agency, Wiltshire, United Kingdom; Fleming Fund Management Agent, Mott MacDonald, London, United Kingdom; Antimicrobial Resistance Coordinating Centre, Ministry of Health, Lilongwe, Malawi; Public Health Institute of Malawi, Lilongwe, Malawi; ESA Regional Hub, Mott MacDonald, Kampala, Uganda; Fleming Fund Country Grant to Indonesia, DAI, Jakarta, Indonesia; Infectious Diseases Research Unit, GSK HQ, London, United Kingdom; Itad Ltd, Brighton, United Kingdom; Family Health and Infectious Diseases, East Central and Southern Africa Health Community, Arusha, Tanzania; Department of Pharmacy, Makerere University School of Health Sciences, Kampala, Uganda; Department of Women's and Children's Health, International Child Health and Migration, Uppsala University, Uppsala, Sweden; Fleming Fund Management Agent, Mott MacDonald, London, United Kingdom; Fleming Fund Management Agent, Mott MacDonald, London, United Kingdom; Department of Medical Microbiology, University of Ghana Medical School, University of Ghana, Accra, Ghana; Itad Ltd, Brighton, United Kingdom; Department of Health Sciences, Vrije Universiteit Amsterdam, Amsterdam, The Netherlands; LAMP Development Organisation, Edinburgh, United Kingdom; College of Medicine and Allied Health Sciences, University of Sierra Leone, Freetown, Sierra Leone; National Public Health Agency, Ministry of Health, Freetown, Sierra Leone; Itad Ltd, Brighton, United Kingdom; Oxford Health Evaluations, Buckingham, United Kingdom; Monitoring and Evaluation, South Asia, Mott MacDonald, New Delhi, India; Itad Ltd, Brighton, United Kingdom; IDS, Mott MacDonald, Noida, India; Central Department of Microbiology, Tribhuvan University, Kathmandu, Nepal; Department of Health and Social Care, London, United Kingdom; Fleming Fund Country Grant to Pakistan, DAI, Islamabad, Pakistan; Regional Office for Asia and the Pacific, Food and Agriculture Organization of the United Nations, Bangkok, Thailand; Department of Veterinary Public Health and Epidemiology, Federal Ministry of Livestock Development, Abuja, Nigeria; Department of Health and Social Care, London, United Kingdom; The School of Medicine, The University of St Andrews, St Andrews, United Kingdom; Department of Clinical Sciences, Liverpool School of Tropical Medicine, Liverpool, United Kingdom; Tāwharau Ora, School of Veterinary Science, Massey University, Palmerston North, New Zealand; Antimicrobial Resistance, International Vaccine Institute, Seoul, Republic of Korea; Fleming Fund Country Grant to Bangladesh, DAI, Dhaka, Bangladesh; Cambodia Oxford Medical Research Unit, Angkor Hospital for Children, Siem Reap, Cambodia; Centre for Tropical Medicine and Global Health, Nuffield Department of Medicine, University of Oxford, Oxford, United Kingdom; Science, International Center of Antimicrobial Resistance Solutions, Copenhagen, Denmark; Fleming Fund Country Grant to Pakistan, DAI, Islamabad, Pakistan; Itad Ltd, Brighton, United Kingdom; Independent Consultant, United Kingdom; STRIDES Nepal Project, Kathmandu, Nepal; Policy & Economic Research, International Vaccine Institute, Seoul, Republic of Korea; Itad Ltd, Brighton, United Kingdom; Institute of Development Studies, Brighton, United Kingdom; Centre for Tropical Medicine and Global Health, Nuffield Department of Medicine, University of Oxford, Oxford, United Kingdom; Department of Microbiology, Lao-Oxford-Mahosot Hospital-Wellcome Trust Research Unit, Vientiane, Lao PDR; Fleming Fund Country Grant to Pakistan, DAI, Islamabad, Pakistan; Itad Ltd, Brighton, United Kingdom; Independent Consultant, United Kingdom; Fleming Fund Country Grant, University of North Carolina Project Malawi, Lilongwe, Malawi; Fleming Fund Management Agent, Mott MacDonald, London, United Kingdom; Disease Prevention and Health Promotion, Nigeria Centre for Disease Control and Prevention, Abuja, Nigeria; Community Medicine, Veritas University, Abuja, Nigeria; Fleming Fund Management Agent, Mott MacDonald, Bangkok, Thailand

## Abstract

**Background:**

Drawing on the Fleming Fund's work to strengthen antimicrobial resistance (AMR) surveillance across Africa and Asia during 2018–2026, this article provides one of the first syntheses of AMR surveillance data use in low- and middle-income countries.

**Methods:**

A participatory synthesis for narrative review was conducted to summarize experience across administrative levels (facility, subnational, national, and regional).

**Results:**

Data use was most evident at the facility level, in human health systems where resistance patterns, antimicrobial use, and clinical data increasingly informed antimicrobial stewardship, infection prevention and control, and other quality improvement initiatives. Data use in other sectors required different approaches. At the national level, Fleming Fund investments were associated with revisions of treatment guidelines, updates of essential medicines lists, and regulatory measures, primarily through improved governance and policy communities. Regional platforms supported member country data use at the national level. Subnational data use was undertaken in countries with decentralized health systems. Across levels, data use pathways driven by local priorities helped surveillance inform action.

**Conclusions:**

Fleming Fund experience suggests that surveillance is more likely to inform action when data use is built into system design. Continued investment will be needed if AMR surveillance is to improve policy and practice in low- and middle-income countries.

This article is part of a journal series synthesizing the evidence and lessons from the Fleming Fund. It contributes to wider learning on how complex global health programs translate technical capacity into institutional and policy change. It also aims to strengthen the use of evaluative approaches within antimicrobial resistance programming and the wider literature.

The purpose of public health surveillance systems is to use data to inform decisions and action [1]. The Fleming Fund worked with low- and middle-income countries (LMICs) across Africa and Asia from 2018 to 2026 to establish and strengthen surveillance systems to inform action against the growing threat of antimicrobial resistance (AMR) [2]. The program responded to the 2015 World Health Organization (WHO) Global Action Plan on AMR, which positioned AMR surveillance as a key pillar to “strengthen the knowledge and evidence base” [3].

Addressing initial conditions in which “surveillance data [were] lacking” for LMICs [4], phase 1 of the program, over 2018–2023, focused on establishing quality-assured microbiology capacity to generate actionable AMR data [5]. As anticipated in the program design, phase 2 implementation over 2023–2026 shifted investment toward data use as data availability increased. Relevant spending increased from around 19% in phase 1 investment plans (14% for “building the enabling environment” and 5% for “advocacy & data utilization”) to 29% of direct costs overseen by the Management Agent in phase 2 (7.6% to “analyze data,” 11.9% to “share data,” and 9.6% to “sustain”) [2, 6].

Figure 1 is a simplified representation of how the Fleming Fund conceptualized data use, based on the program's theory of change [2]. Data—typically from antimicrobial susceptibility testing (AST)—were a core input to surveillance, including analysis, data sharing and institutional development. Action was normally framed in terms of either policy or practice, mainly to distinguish between government policy initiatives and professional practice or behavior. The aim was for data to inform both policy and practice over time, including during a planned phase 3 that was never funded.

**Figure 1.**
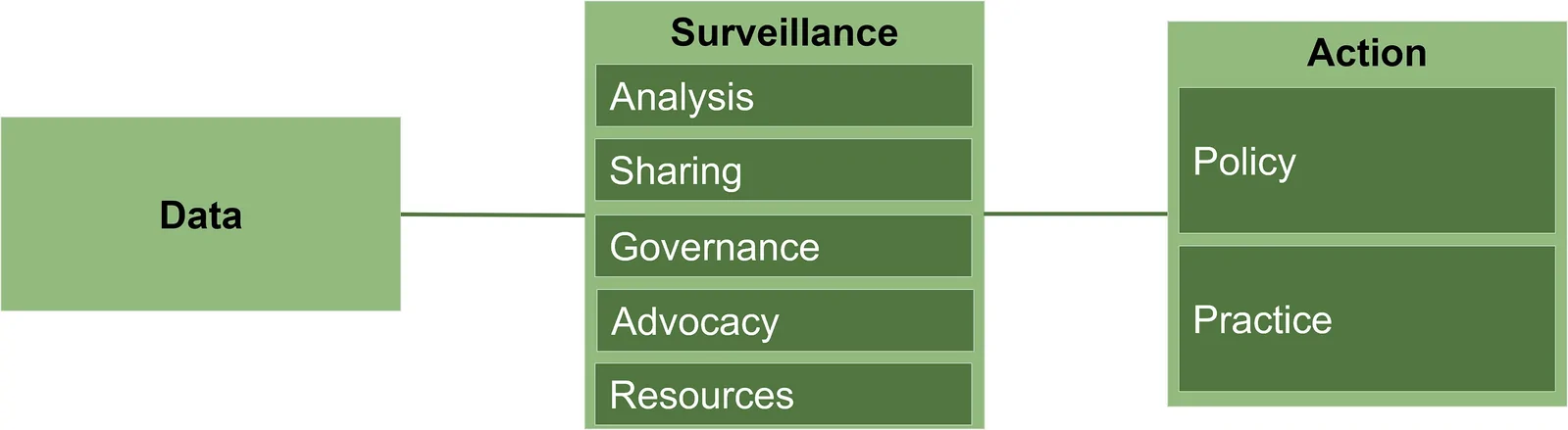
Representation of Fleming Fund conceptualization of data use.

Action on AMR was defined in the 2015 Global Action Plan, and consequently in many countries’ AMR national action plans (NAPs), in terms of efforts to “optimize the use of antimicrobial medicines” (antimicrobial stewardship [AMS]) or to “reduce the incidence of infection through effective sanitation, hygiene and infection prevention measures” (infection prevention and control [IPC]). These objectives translate into a wide range of possible actions across contexts, in view of the “One Health” nature of AMR (as an agenda that recognizes the importance of environmental and animal as well as human health) [7]. Figure 2 lists some possible actions against AMR, showing how these take different forms across sectors.

**Figure 2.**
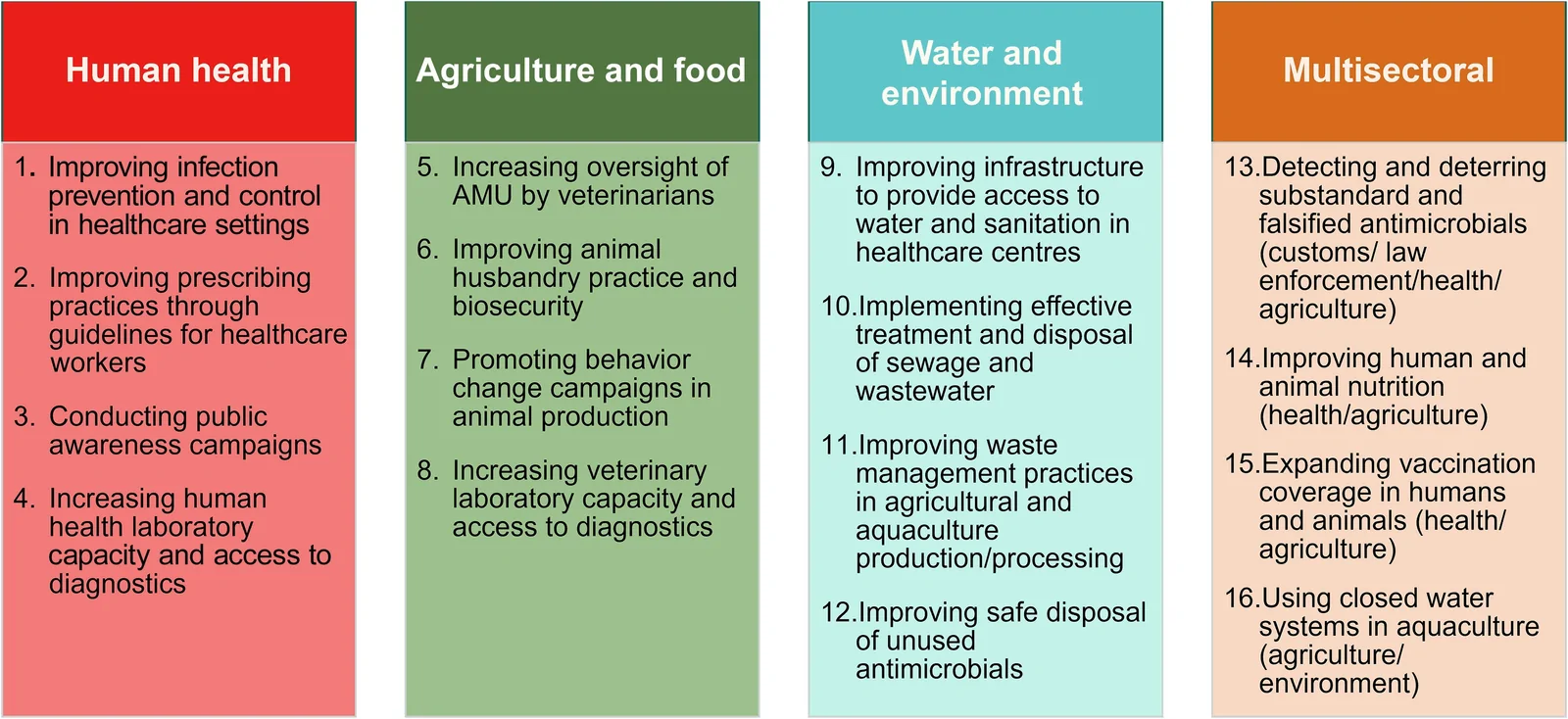
Possible actions against antimicrobial resistance across sectors (drawn from World Bank “intervention areas” [8]). Abbreviation: AMU, antimicrobial use.

Surveillance interventions implemented by the Fleming Fund were guided by a scoping study that concentrated on laboratory capacity (action 4 in Figure 2) as a basis for surveillance system strengthening [9]. The program followed a road map for the participation of LMICs in the Global Antimicrobial Resistance and Use Surveillance System, which recommended establishing and gradually expanding networks of sentinel sites [10].

Various program elements were included to address data use in LMICs. Data use elements from phase 1 onward included support for country governance frameworks [11], “Policy Fellows” under the Fleming Fellowship Scheme [2, 12], and the Regional Antimicrobial Resistance Data Analysis for Advocacy, Response and Policy (RADAAR) project [13]. Additional elements were added for greater focus on data use in phase 2: grantees were required, as part of their contracts, to prioritize data use; and additional regional grants mobilized technical assistance for “data and evidence use” (TADEU) and technical assistance for “clinical engagement” (TACE) for each of Africa and Asia [14].

Although the Fleming Fund was a substantial global investment, allocations per country were “relatively modest” for the range of activity undertaken over the time scales involved (ranging from a grand total of about £15 million in Nigeria to about £2 million in Rwanda). Financial information at program close suggests that the dominant categories of spending specifically on data use in phase 2 were “data analysis carried out” and “surveillance reports used to inform practice” [6].

Different actions and data use pathways require different data types. Fleming Fund investments focused on AMR data quantity and quality (ie, data from antimicrobial susceptibility testing). However, the program also invested in data on antimicrobial use (AMU), antimicrobial consumption, burden of disease (ie, demographic and outcome data), and to enable economic analysis (including estimates of the costs and benefits of surveillance and different options for action).

The Fleming Fund supported generation of a very substantial amount of quality data, including the processing about 7 million samples collected from human patients and >400 000 in animal health, supporting >300 sites across the world [5].

This article provides one of the first multicountry, multilevel syntheses of how surveillance data are used to inform decision making and action on AMR in LMICs. Recent guidance from Thailand notes that the “challenge of how to use AMR surveillance data for actions in LMICs still lacks research and evidence” [15].

## METHODS

This article forms part of a practice-informed series synthesizing learning from Fleming Fund–supported AMR surveillance strengthening activities. It draws on program documentation, evaluation evidence, relevant literature and structured engagement with a broad range of contributors from multiple disciplines and perspectives, including implementing countries and the wider AMR community. The series does not constitute an independent evaluation or seek to establish causal attribution. Findings should therefore be interpreted in the light of limitations, including program-linked authorship, contributor perspectives, and purposive source selection. Further methodological detail is provided elsewhere in this series [2].

The long list of potential authors for this article included 99 people, with 48 choosing to participate. A short survey at the outset received 36 responses (75%), confirming broad familiarity across Fleming Fund countries and regions, data use levels, surveillance/action types, and evidence perspectives. 23 of the authors (48%) are women.

A targeted literature review was undertaken to add to examples suggested by authors. The review defined data use experience in terms of surveillance to drive stewardship/prevention in any One Health Sector and focusing on data generated in the surveillance system, including laboratory/microbiology data on drug resistance, as well as antibiotic use and consumption and explicitly focusing on Fleming Fund–related material (program publications and gray literature as well as academic publications). Sources included a database of program products and publications compiled by the management agent for the United Kingdom Department of Health and Social Care in September 2025. One specific limitation for this article was that the lack of established frameworks or benchmarks for data use in AMR surveillance obstructed quantitative assessment.

Insights are presented across 4 levels—facility, national, regional, and subnational—consistent with routine health system administrative classifications. See Table 1 for indicative examples. Note that the levels are presented in a nonintuitive order, according to the level of priority in Fleming Fund implementation. Also note that Table 1 is not a comprehensive or causal representation; caution should be exercised when making any assumptions about the relationship between levels. Global-level data use was excluded to avoid overlap with the article by Chandler et al [7] elsewhere in this series.

**Table 1.** System Levels Used for this Article, With Indicative Examples From Fleming Fund Work in Human Health Systems

Level	Institutional Focus	Data Example	Example of Surveillance Activity	Action Example	Data Use Examples in Human Health Systems^a^
Facility	Hospital microbiology laboratory; animal health laboratory	AST results (AMR)	Analysis of AMR patterns (antibiogram)	Local antibiotic prescribing guidelines	AMR Control Working Group initiatives at Queen Elizabeth Central Hospital in Malawi [16]; targeted antibiotic therapy for neonatal sepsis at Hospital Nacional Guido Valadares in Timor-Leste [17]
National	AMRCC; national drug regulator	Border data on antimicrobial import volumes (AMC)	Sharing from customs authority to national drug regulatory authority	Prohibition of certain molecules	Nepal Department of Drug Administration prohibits “not recommended” antimicrobials, 2023 [18]; Zambia Ministry of Health revises national EML, 2025 [19]
Regional	Regional technical center	Series of PPSs (AMU)	Governance initiative for peer-to-peer learning	Recommendations for professional education curricula	Africa Centres for Disease Control and Prevention (Africa CDC) AMR surveillance guidance [20]
Subnational	District or provincial authorities	Cost estimates for diagnostic services (economic)	Advocacy for public budget allocation	Investment in diagnostic services	Costing AMR surveillance program in Sindh Province, Pakistan [21]

Abbreviations: AMC, antimicrobial consumption; AMR, antimicrobial resistance; AMRCC, Antimicrobial Resistance Coordinating Committee; AMU, antimicrobial use; AST, antimicrobial susceptibility testing; EML, essential medicines list; PPSs, point prevalence surveys.

^a^See also the case study of Fleming Fund achievements in Kenya elsewhere in this series, including data use achievements at various levels [22].

## FACILITY LEVEL: ANCHORING AMR SURVEILLANCE IN PATIENT OUTCOMES

### Guiding Treatment for Individual Patients

At the facility level, AMR data guide antibiotic treatment for individual patients. Health facilities can generate substantial antimicrobial susceptibility testing data from routine diagnostic testing of samples from patients with suspected infections. (The human health surveillance component of the Fleming Fund was primarily based on culturing blood and other clinically relevant samples of sick individuals presenting to the hospital. A blood culture that yields a typical pathogen in the context of a sick individual likely represents a bloodstream infection or even a diagnosis of sepsis.) These data support surveillance, particularly when aggregated into antibiograms (a summary of the proportion of bacterial isolates susceptible to different antibiotics) and used to inform local empiric antimicrobial prescribing guidelines [23]. In LMICs, strengthening laboratories can therefore directly improve patient care [24], with surveillance data as a byproduct (already widely available in many high-income settings due to stronger clinical microbiology services). Supporting clinical decision making with microbiology data has been shown to be a cost-effective intervention for reducing morbidity and mortality rates [17, 25].

### Interactions Between Microbiology Laboratories and Clinical Functions

The Fleming Fund’s approach to facility-level data use emphasized interactions between microbiology laboratories and clinical functions, from the outset but especially in phase 2. Frontline clinical engagement was expected to create a feedback cycle between microbiology laboratories and clinical services. In this model, microbiological data inform clinical practice improvement initiatives, which in turn are expected to increase demand for microbiology services and for aggregated surveillance data. Effective clinical engagement required active involvement of local leadership in Fleming Fund investments. Fleming Fund efforts supported the establishment of AMS, IPC, and/or diagnostic stewardship committees in participating sites, with support from country grants, the Surveillance and Prescribing Support for Antimicrobial Stewardship Resource Capacity Building (SPARC) project, and also TACE grants in Asia and Africa [14]. Monitoring data showed a substantial increase in data sharing with local stakeholders, a 2-fold increase in data sharing with AMS committees, and a 3-fold increase in data sharing with IPC committees over 2024–2025 [26]. Independent evaluation analysis of the same site-reporting data showed that the numbers of Fleming Fund–supported human health sentinel sites reporting local data sharing doubled over 2021–2025 (from <75 to >150 sites) [6].

### Need for Local Clinical Data

Local clinical data were also needed to inform facility-level action. Some hospitals reported action in response to spikes in drug-resistant infections. At the National Referral Hospital in Bhutan, the hospital-acquired infection rate decreased from 12.3% in 2024 to 6.9% in 2025 in the context of active IPC and AMS involvement and establishment of an AMR/multidrug-resistant organism alert group [27]. Other examples of hospitals that produced antibiograms and/or developed facility-level antibiotic treatment guidelines based on local resistance patterns were documented [28–30]. Additional efforts strengthened surveillance systems and data integration (integration of laboratory with health information systems), and enhanced local staff capabilities and mandates for analysis and interpretation of data to inform priority actions.

### AMU Data

AMU Data also informed facility-level action. There was an increase in the number of hospitals that undertook ≥1 point prevalence survey of AMU and AMR patterns to inform practice (for example [31–33]). The Fleming Fund SPARC program combined point prevalence surveys with continuous quality improvement initiatives [34]. The Commonwealth Partnerships for Antimicrobial Stewardship program also emphasized AMU data use as a driver of stewardship action through facility partnerships [35].

### Practical Constraints

Facility-level data use faced practical constraints [36]. Clinical engagement implies some level of local resourcing for a clinical microbiology or liaison service and for work to aggregate data to inform guidelines. Clinical improvement initiatives were pursued as health worker behavior change efforts (eg, with hand hygiene and handwashing), normally requiring long-term support over multiple cycles to effect and sustain change [37]. Bearing in mind the severity and persistence of various types of local resource constraint in LMICs, including lack of access to appropriate antibiotics of acceptable quality [38], Fleming Fund and other interventions on AMR surveillance in LMICs increasingly focused on developing pragmatic, minimal-resource approaches, standards for surveillance and action, such as program monitoring of local data sharing (see above), A Clinically Oriented Antimicrobial Resistance Network (ACORN)–lite [39], and SPARC methods for implementing AMS, with less reliance on regular formal point prevalence surveys.

### Current State of the Evidence on Outcomes

The evidence of improved health outcomes from data use at the facility level remains unclear, and the case for investment inconclusive pending further investigation in operational settings. Health outcome monitoring was constrained by wider challenges in formalizing and implementing action initiatives in LMICs, including limited staffing, competing clinical priorities and the absence of routine clinical outcome measurement systems or benchmarks. Under these circumstances, program monitoring had to focus on process activity rather than outcomes [26]. The program could therefore not test or validate positive feedback effects from clinical engagement. This had especially severe implications for justifying local resourcing and therefore for sustainability. The intention was for data use to demonstrate the value of facility-level surveillance to hospital managers at modest additional cost beyond that of microbiology services for patient treatment. Although anecdotal evidence suggests such effects in some sites, evidence of improved local resource allocation remains limited.

### Expansion of Facility-Level Data Use Beyond Hospital Settings

In human health systems, Fleming Fund pharmacy-led partnerships in Africa shifted over time to emphasize AMU and consumption (AMU/C) data use in primary care and community settings, including through a “hub and spoke” model leveraging local leadership for systematic change [40, 41]. Broader understandings of AMR action at facility level promoted awareness-oriented approaches alongside knowledge-based professional efforts. Newer efforts such as electronic medicines management systems bridge consumption surveillance and community-based action on AMS [42]. These remain small-scale and early efforts to address entrenched behavioral and market drivers of AMR.

### Animal Health, Food Safety, and Environmental Health Laboratories

Data use around animal health, food safety and environmental health laboratories was different from data use in human health systems at the facility level. Absolute levels of investment in laboratory systems in these sectors are typically much lower than in human health, making surveillance more reliant on program investments like the Fleming Fund [43]. Outside of human health, surveillance more often requires additional data collection through active surveillance (seeking out samples) in combination with passive or sentinel surveillance (exploiting data flows from routine clinical practice) [44]. Local end users of laboratory services in these sectors are also less cohesively grouped, at least beyond concentrated industry clusters, and surveillance efforts are more immediately confronted with a need to engage the private sector and paraprofessional or nonprofessional communities.

Animal health surveillance helped show where AMR approaches needed to be adapted to specific species, production systems, and husbandry practices [45], including a focus on poultry rearing and aquaculture as AMR “hot spots.” Data use was most evident in participatory, action-oriented approaches involving AMU/C data, including Farmer Field School methods [46, 47]. Fleming Fund local data use initiatives beyond human health also encouraged closer attention to wastewater [48] and abattoir monitoring [49] as opportunities for local data use from AMR surveillance. Although there were instances of local multisectoral data use initiatives under the Fleming Fund, for example in Uganda [50], facility-level data use remained mainly sector-specific.

## NATIONAL LEVEL: ENABLING ACTION THROUGH POLICY INSTRUMENTS

### National Initiatives

Beyond the frameworks in AMR NAPs, certain initiatives have emerged in recent years as recurring national-level actions on AMR in Fleming Fund–supported countries [51]. Relevant national instruments included: (1) national standard treatment guidelines to guide rational prescribing; (2) the national essential medicines list to guide supply chain decisions, including in line with the WHO Access, Watch, and Reserve (AWaRe) classification [52]; and (3) exercise of national drug regulators’ existing market control powers to enact measures prohibiting certain antimicrobials, limiting specific uses, or mandating improved commercial pharmacy practices.

Kenya illustrates these forms of national action (see Wesangula et al [22] elsewhere in this series), as does Nepal [18], among other examples. Phase 2 supported the standard treatment guidelines and the national essential medicines list directly in the animal health sector, for example in Bangladesh [53].

### Policy Communities and Institutions

National-level data use was supported by stronger policy communities and institutions involved in AMR action. Country grants included governance activities, such as support to NAP development and Antimicrobial Resistance Coordinating Committee (AMRCC) functions [2]. The program empowered relevant technical networks across policy and practice (diagnostics, clinical microbiology, and ministry functions, including AMRCC functions themselves). As well as training and education initiatives under country grants and communities of practice developed by the RADAAR project, relevant program interventions included the Fellowship Scheme [12] and the Qualifying the Workforce for AMR Surveillance project [54]. Communities of practice were formed, for example in Nigeria [55]. Surveillance was also linked to advocacy and awareness-raising, including through the World AMR Awareness Week. A recent difference-in-difference study using self-reported data from the Tracking AMR Country Self-Assessment Survey (TrACSS) indicated that Fleming Fund countries have made better progress than other LMICs across a range of country surveillance governance indicators, including use of surveillance data in decision making [56].

### AMR Data as a Resource

The Fleming Fund helped position AMR data as a resource for national-level action, especially following increased attention to data use in phase 2, building on data use initiatives at the facility level (see above). National AMR dashboards [57] are increasingly available for monitoring spatial and temporal trends, for example in Bangladesh and Laos [58, 59], as well as in Kenya. Fleming Fund support contributed to the production of national antibiograms for the first time in some countries, for example, Nepal [60]. It was also increasingly possible to mobilize AMU data at the national level, for example, in Laos and Sierra Leone [59, 61].

### Strengthening of Laboratory Systems Capacity

The extent to which strengthening laboratory-systems capacity encouraged national-level action varied by context. In settings previously lacking substantial AMR data, it proved easier to explain the direct relevance of laboratory systems capacity investment to policy or regulatory action. The contribution of laboratory systems capacity strengthening was more compelling in Africa, with resourcing gaps observed by the Mapping Antimicrobial Resistance and Antimicrobial Use Partnership [62]. Existing laboratory systems capacity was generally stronger in Asia, with historical data availability reported by the Capturing Data on Antimicrobial Resistance Patterns and Trends in Use in Regions of Asia (CAPTURA) (gathering 2.3 million antimicrobial susceptibility testing records from 72 laboratories across 7 countries in South and South-East Asia through stakeholder engagement and collation) [63]. In some countries, emphasis on data generation may even have delayed action while data accumulated [64]. Greater scope for differentiation according to country and institutional context has been suggested as a major lesson from the Fleming Fund evaluation [51].

### Data Limitations

Data generated alone were often insufficient to support national decision making. Use of clinical diagnostic specimens is known to introduce a risk of bias because results are not representative of populations, although the approach has been adopted internationally and is considered especially suitable for LMICs because it is relatively easy to implement and sustain [2, 25]. These limitations were recognized from the outset and are inherent to sentinel and routine diagnostic-based surveillance systems globally, which rely on nonrandom samples and are therefore not fully representative of the underlying population. AMR data, the primary focus of Fleming Fund design from the outset, indicate the scale of the problem. The program therefore increased relative emphasis over time on AMU/C, burden and other data that are more relevant to justifying and monitoring progress toward solutions. Consumption surveillance emerged as a potentially important and relatively achievable basis for regulatory action at national level in some countries (eg, Bangladesh, Nepal, and Nigeria).

### Investment in Data Use

Investment in data use intensified once data volume and quality improved, although context-sensitive evidence translation expertise remained underdeveloped within the available time frame. A special supplement in *Clinical Infectious Diseases* in 2023 included an article setting out key recommendations from the Fleming Fund CAPTURA project in Asia, noting a “huge gap in data use” including to inform national policy [65]. Various challenges kept the “evidence-to-policy” gap wide in LMICs, despite increased attention in phase 2 [66]. Where national-level policy change occurred, it was often based not on locally-generated data and evidence but on global action—for example, efforts to phase out veterinary uses of colistin [67]. Program implementation naturally tended to focus on uses of the data generated through program investments, giving less attention to nondata factors, research, and evidential inputs from the global level (not discussed in this article, such as standards, research, exemplars, and action targets, as well as feedback from global surveillance).

### Focus on Priority Actions

In phase 2, national-level data use increasingly focused on an “action-oriented prioritization” approach, focusing on “data that have the highest likelihood of affecting change that can achieve impact” [68]. The Smart Choice Process for NAP targeting has helped establish action priorities in various African countries [69]. Action-oriented prioritization takes existing country agendas as the starting point for AMR surveillance and data system design, rather than “data driven” aims relying on motivation arising from the data. A 2026 Fleming Fund case study review on surveillance in animal health recommended a “scan and target” approach to sampling, working across food safety and wastewater as well as veterinary systems to adapt to varying country institutional landscapes [70]. The RADAAR project's phase 2 dialogue processes also applied good practice in participatory evidence-based policy development to promote partner country ownership and commitment to focused AMR action in Bhutan, Ghana, Kenya, and Laos [66, 71].

### The Economic Case for Action

The “economic case” for action against AMR was increasingly seen as important for policy advocacy over the program implementation period, with various options for surveillance initiatives presenting different cost profiles [17, 72, 73] and requiring justification based on cost-benefit analysis of the actions they are designed to motivate [74, 75]. In the context of affordability and sustainability concerns, program economic analyses mainly focused on laboratory investments as a key system cost driver, rather than using economic evidence on the wider data use landscape.

### Sustainability

The sustainability of surveillance improvements achieved by the Fleming Fund will likely depend on whether they are useful to domestic agendas, with strong leadership and demand in countries’ own political economy contexts [76]. National health insurance system coverage of relevant diagnostic testing as part of universal health coverage efforts is increasingly seen as an important determinant [21]. AMR surveillance can be usefully integrated into wider efforts to improve access to diagnostic services for bacterial infections, help track and inform clinical management of infections in health systems, and develop public health surveillance and/or emergency prevention, preparedness, and response plans [77]. In the animal health sector, the AMR action agenda can also usefully align with national agricultural extension and export promotion, as well as disease prevention efforts. AMR surveillance and data use should be included in national livestock development agendas or broader agricultural and food security plans [78]. This relies on the surveillance system producing sector-relevant data (see further discussion elsewhere in this series [7, 79]).

### Benchmarks for Effective AMR Surveillance Data Use

There are opportunities to improve benchmarks for effective AMR surveillance data use at national level, drawing on Fleming Fund experience to adapt existing frameworks. Currently the TrACSS includes 1 question on the use of data to inform decisions [6, 56], but guidance on monitoring of NAP implementation treats strengthened knowledge and evidence as an outcome alongside action (rather than as a factor contributing to action) [79]. Better measurement and demonstration of surveillance data use at the national level might involve clear and explicit specification of which national policies, decisions, and/or actions AMR surveillance is intended to inform, and how. Future research might usefully investigate appropriate benchmarks for national data use involving decision-maker feedback, for example, from technical working groups or committees formed to revise the standard treatment guidelines or the national essential medicines list.

## REGIONAL LEVEL: ENCOURAGING AND SUPPORTING NATIONAL ACTION

### Peer Learning and Technical Exchange

Regional organizations and initiatives supported national-level data use through peer learning and technical exchange. Peer influence and other regional factors may help explain the cross-country variation in policy and regulatory initiatives observed in the summative evaluation of the Fleming Fund [51]. Regional bodies like the East Central and Southern Africa Health Community, the Africa CDC (See Table 1), the Association of Southeast Asian Nations, and Quadripartite regional offices promoted strategies and approaches for action on AMR [81–84]. (The Quadripartite is a collaborative partnership of 4 international organizations focused on implementing a "One Health" approach to manage health risks at the human-animal-environment interface. The 4 organizations are the Food and Agriculture Organization of the United Nations, the United Nations Environment Programme, WHO, and the World Organization for Animal Health.)

### Professional Standards and Technological Approaches

The regional level supported professional standards and advanced technological approaches. In Africa, the African Society for Laboratory Medicine played an important role in workforce development through the Qualifying the Workforce for AMR Surveillance grant and in policy community development through the TADEU grant for Africa. Fleming Fund activities to develop genomic surveillance were also organized on a regional basis, although there is no evidence yet of genomic data influencing policy or practice [85].

### Promoting Action in Regional Forums

More could be done to promote action on AMR through data use in regional forums. A series of RADAAR project studies in 2021 highlighted the advantages of making better use of the regional level in AMR surveillance [86]. There is special interest in pursuing antimicrobial consumption monitoring in South-East Asia [87].

## SUBNATIONAL LEVEL: CONNECTIONS BETWEEN FACILITY AND NATIONAL LEVELS

### Importance in Larger Countries and Decentralized Systems

Data use at the subnational level was especially important in larger countries and in those with decentralized health systems. Implementers in larger, federal countries faced greater challenges and resource demands to pursue data use. In Pakistan, Fleming Fund initiatives that were pursued at the national level in other countries were necessarily implemented as collaborations with provincial governments—for example, an antibiogram in Punjab and an AMR dashboard in Gilgit-Baltistan [88, 89].

### Linkage Between National- and Facility-Level Data Use

Subnational data use linked national- and facility-level data use to action. In Kenya, scaling across counties and working with increasing numbers of County Antimicrobial Stewardship Interagency Committees was a major feature of Fleming Fund success [90] (see also Wesangula et al [22] elsewhere in this series for more detail). The “hub and spoke” approach mentioned above also relies on subnational frameworks. From a national data use perspective, key national actions (treatment guidelines and essential medicines lists) operate as enabling and supporting inputs intended to guide local action. Contrary to viewing national actions as potential higher-level results, this suggests that they are better understood as starting points, or inputs, for data use interventions.

### Utility of Additional Initiatives at the Subnational Level

The utility of additional data use initiatives at the subnational level became increasingly apparent over the course of Fleming Fund implementation because of decentralized resource allocation decision making. Sustainability analyses developed over phase 2 from an initial focus on estimates for national budget allocations [91]. AMR surveillance resourcing came to be seen as a question of prioritization within existing sector, program, locality or facility allocations [21].

## DISCUSSION: FLEMING FUND EXPERIENCE OF DATA USE ACROSS LEVELS

### Relative Emphasis on the Facility Level

The Fleming Fund placed relatively strong emphasis on data use at the facility level and action in terms of clinical practice. Global expectations for surveillance data use currently focus on national level (see figure from the Quadripartite Joint Secretariat) [84]. The program instead pursued data use at the facility level with equal or greater determination than at the national level. A design shaped by global surveillance goals guided investment into networks of surveillance sites, which then required a focus on facility-level data use [7, Figure 3].

### Recognition of Existing Decision Needs

Across levels, the most successful data use pathways started from existing decision needs. Study of routine clinical data for AMR surveillance shows the need to recognize and address priorities in hospitals and clinical microbiology laboratories before public health objectives, ensuring that interventions prioritize timely data aggregation and use for facility-level purposes over “data capture and onward reporting primarily for surveillance” [23]. At the national level, data systems must also demonstrate their usefulness for country priorities before they can be counted on for global public health purposes.

### Importance of Systems Perspectives

Understanding data use in AMR surveillance initiatives requires systems perspectives, acknowledging the complexity of multifactor interactions across contexts and levels. This includes considering how decisions are made at different levels; the level of priority accorded to each level; challenges as well as opportunities in the interaction between levels; and how financing, incentives, regulation, and professional practice shape what is possible. A flexible approach based on professional networks using multiple data types and action-oriented data use pathways may be more effective than focusing on any single specific data type or pathway, including, for example, antimicrobial consumption data analysis for national-level action, facility-level action in nonhospital settings, and participatory approaches, such as community engagement.

### Wider Factors Affecting Outcomes

Data use outcomes depend on a wide range of factors beyond the control of surveillance systems (or programs like the Fleming Fund, designed to strengthen surveillance systems). Experience of AMR surveillance implementation in LMICs highlights the importance of political, economic, social, legal, and ethical factors in shaping data use. Improved data use depended on governance progress involving various such factors across as well as within levels, underlining the importance of data management and governance frameworks as a significant global issue for the AMR agenda [7]. Data use was more achievable in contexts in which other aspects of AMR action plans were better developed or further progressed, notably, stewardship and IPC programs as part of broader health system strengthening.

### Expectations and Differing Views on Data Use

The expectation that data use would grow over time as data volume, quality, and institutional capacity matured had lasting implications for program effectiveness, especially given program termination. Data use required a profound shift in mindset at both facility and national levels, away from data production and toward user demand as the driving motivation for the program. Program monitoring approaches were designed primarily to track infrastructure, activities, and outputs rather than political or behavioral change. Contracting and implementation requirements limited opportunities for major midcourse adaptation, and plans for continuation into phase 3 were interrupted.

Different stakeholders held differing views on data use (including in debates among the author group during the compilation of this article). Although these perspectives were not fully resolved, implementation evolved toward a broad and inclusive approach to data use in which multiple technical and professional perspectives were recognized as making a valuable contribution.

### Benchmarks for Surveillance Data Use

Improved benchmarks for AMR surveillance data use would better demonstrate the value of surveillance. Fleming Fund activities showed progress on data sharing with decision makers at the facility and national levels (respectively, through program monitoring information and self-reported country TrACSS responses, discussed above). However, extending benchmarks to include systematic measurement of the actions against AMR informed by surveillance data is necessary to demonstrate fully why surveillance should be prioritized in LMICs. Ideally, such benchmarks should also relate surveillance data to national targets for AMR action established in global forums [7].

### Resource Requirements

The resource requirements for data use were greater than initially anticipated at all levels. Data use is not cost neutral; it entails additional and recurrent investments in staff time, systems, coordination, analysis, and decision-making processes beyond those required for data generation. Data use also implies an opportunity cost, as analytical and managerial capacity devoted to AMR surveillance must be weighed against other disease surveillance and public health priorities. While external funding has been relatively effective in supporting laboratory equipment and training, inputs that are comparatively easy to absorb, there is less evidence that externally funded investments in data use can be sustained.

### Challenges

Surveillance data use is challenging and takes time to develop. At the facility level, challenges encountered in linking surveillance to AMS and IPC service improvement efforts warranted greater attention to implementation research to establish what works efficiently. At the national level, the topic was addressed as a technical and scientific challenge, in terms of knowledge translation. Implementers encountered political, sociological and behavioral barriers and made limited use of tools for understanding how to overcome relevant challenges (eg, political economy analysis).

### Operationalizing One Health Principles

Fleming Fund implementation included efforts to operationalize One Health principles, which possibly increased the scope for data use, especially at the national level. Commitment to operationalizing One Health principles is explored in more detail elsewhere in this series [92]. However, there is insufficient evidence to date of systematic benefits from One Health working in terms of data use at any level. There is still limited understanding of how data from one sector might inform action in another, such as national animal health responses prompted by human health surveillance findings. Under these conditions, human health priorities continued to shape data use more strongly than other sectoral perspectives. Sectors are still developing their own understandings of AMR, its drivers and appropriate responses.

## CONCLUSIONS

Surveillance systems are more likely to inform action when data use is built into their design from the outset. Surveillance design should begin by examining the decisions that systems are intended to inform, including at what level(s) and by whom those decisions will be made. Data specifications should follow those decisions rather than precede them, with data then connected to decision making through context-appropriate data use activities. Sustained investment in surveillance also depends on demonstrating value to the people and institutions expected to use the data.

The Fleming Fund’s data use experience suggests 4 main implications for AMR surveillance in LMICs (see Figure 3). First, the case for action on AMR is already established; the role of surveillance is to target, refine, and evaluate action. Second, data alone do not change practice or policy; analysis, interpretation, communication, and institutional uptake are some of the steps that translate surveillance into action. Third, effective AMR action depends on the people and institutions that interpret, negotiate, and act on data within specific contexts. Fourth, sustained investment in data use capacity—not only data generation—is needed if surveillance is to improve practice and policy in LMICs.

**Figure 3.**
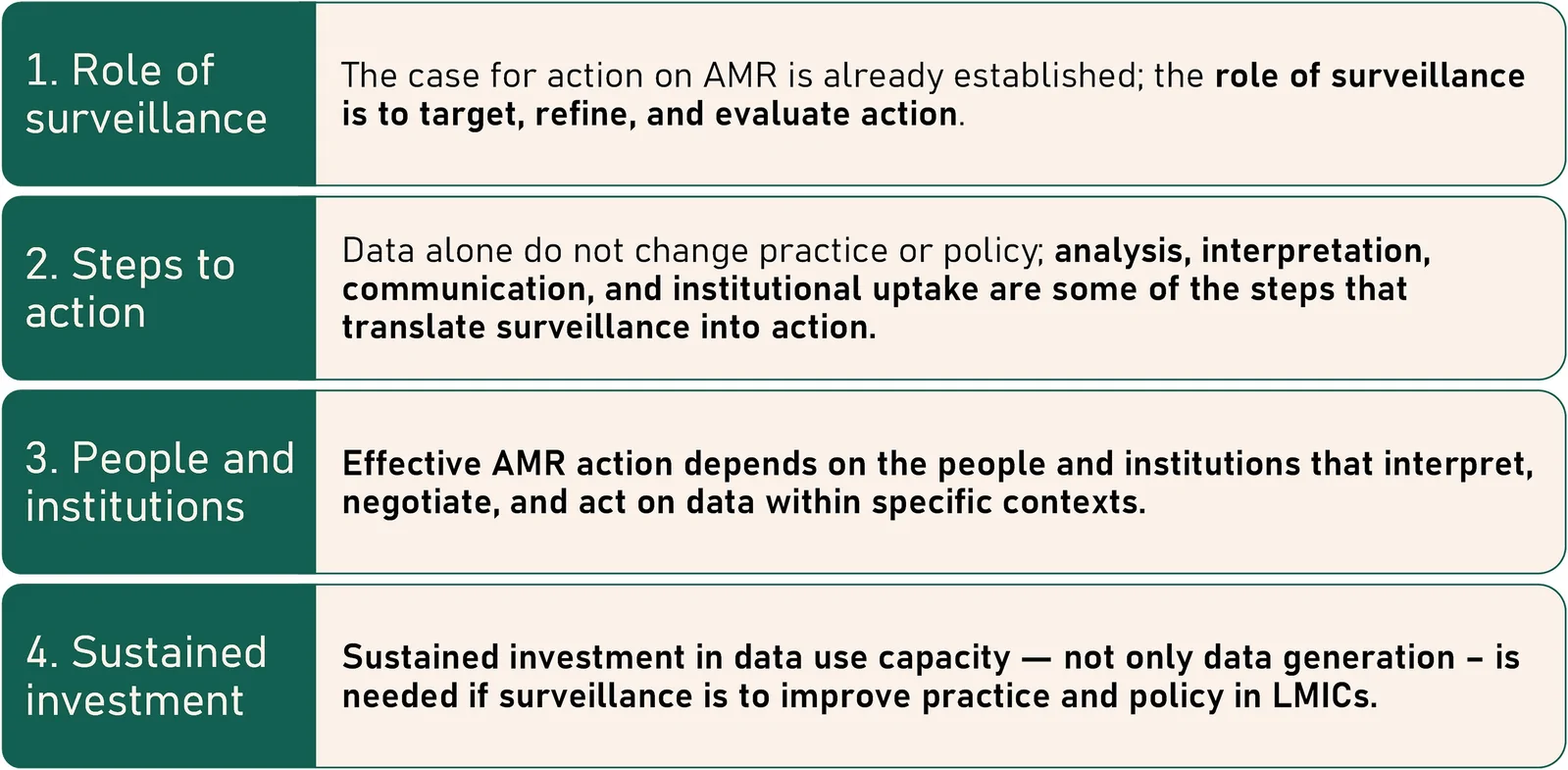
Main implications for antimicrobial resistance (AMR) surveillance in low- and middle-income countries (LMICs) based on Fleming Fund data use experience.

For policymakers and implementers, including officials working on NAP revisions and designers of other surveillance-strengthening programs, the practical implication is that surveillance systems should be designed around priority decisions, with explicit investment in the analytical, governance, and clinical as well as technical capacities required for data use. For funders, support for surveillance should extend beyond data production to the systems and people needed to turn data into action.
